# Molecular Cloning and Functional Analysis of UV RESISTANCE LOCUS 8 (*PeUVR8*) from *Populus euphratica*

**DOI:** 10.1371/journal.pone.0132390

**Published:** 2015-07-14

**Authors:** Ke Mao, Lina Wang, Yuan-Yuan Li, Rongling Wu

**Affiliations:** 1 Center for Computational Biology, College of Biological Science and Technologies, Beijing Forestry University, Beijing, 100083, China; 2 College of Horticulture Science and Engineering, Shandong Agricultural University, Tai-An, Shandong, 271018, China; 3 Center for Statistical Genetics, The Pennsylvania State University, Hershey, Pennsylvania, 17033, United States of America; University of Vigo, SPAIN

## Abstract

Ultraviolet-B (UV-B; 280–315 nm) light, which is an integral part of the solar radiation reaching the surface of the Earth, induces a broad range of physiological responses in plants. The UV RESISTANCE LOCUS 8 (UVR8) protein is the first and only light photoreceptor characterized to date that is specific for UV-B light and it regulates various aspects of plant growth and development in response to UV-B light. Despite its involvement in the control of important plant traits, most studies on UV-B photoreceptors have focused on Arabidopsis and no data on UVR8 function are available for forest trees. In this study, we isolated a homologue of the UV receptor UVR8 of Arabidopsis, *PeUVR8*, from *Populus euphratica* (Euphrates poplar) and analyzed its structure and function in detail. The deduced PeUVR8 amino acid sequence contained nine well-conserved regulator of chromosome condensation 1 (RCC1) repeats and the region 27 amino acids from the C terminus (C27) that interact with COP1 (CONSTITUTIVELY PHOTOMORPHOGENIC1). Secondary and tertiary structure analysis showed that PeUVR8 shares high similarity with the AtUVR8 protein from *Arabidopsis thaliana*. Using heterologous expression in Arabidopsis, we showed that *PeUVR8* overexpression rescued the *uvr8* mutant phenotype. In addition, *PeUVR8* overexpression in wild-type background seedlings grown under UV-B light inhibited hypocotyl elongation and enhanced anthocyanin accumulation. Furthermore, we examined the interaction between PeUVR8 and AtCOP1 using a bimolecular fluorescence complementation (BiFC) assay. Our data provide evidence that PeUVR8 plays important roles in the control of photomorphogenesis in planta.

## Introduction

Sunlight is important to plants both as the primary source of energy and as an environmental signal that regulates growth and development. Light promotes the developmental transition from skotomorphogenesis to photomorphogenesis in plants through the combinatorial interaction of diverse sensory photoreceptors, which are classified based on the wavelength of light they perceive [1]. Light signals are perceived through at least five distinct families of photoreceptors: red/far-red (600–750 nm) light receptor phytochromes [2]; blue/UV-A (315–500 nm) light receptor phototropins [3], cryptochromes [4,5], and the F-box proteins ZEITLUPE (ZTL), FLAVIN-BINDING KELCH REPEAT F-BOX1 (FKF1), and LOV KELCH PROTEIN2 (LKP2); and the UV-B (280–315 nm) light receptor UV RESISTANCE LOCUS 8 (UVR8) [6].

UV-B wavelengths impinge on the earth with highly variable spatial and time-dependent distributions [7]. While UV-B light is a source of damage, it is also a source of information for plants. At the physiological level, UV-B light causes altered flowering time, promotion of branching, reduced fertility, and reduced biomass production [8,9]. UV-B light responses are dependent on the fluence rate and can be divided into a stress response at damaging UV-B fluence rates and an acclimation response at non-damaging UV-B fluence rates [10,11,12]. Non-damaging UV-B light evokes photomorphogenic responses including hypocotyl growth inhibition, cotyledon expansion, phototropic curvature, biosynthesis of anthocyanins and flavonoids, and stomatal opening [13,14,15,16,17].

To optimize their growth and survival, plants perceive and respond to UV-B radiation. The molecular identity of the UV-B photoreceptor and the photoperception mechanism were unknown until Rizzini et al. showed that the protein that perceives UV-B light was the β-propeller protein UVR8 [6]. *Arabidopsis* UVR8 is a 440-amino-acid protein consisting of a highly conserved seven-bladed β-propeller core with a short N-terminal extension and an apparently flexible C-terminal region of approximately 60 amino acids [18]. Sequence analysis showed that UVR8 shares sequence similarity with the human guanine nucleotide exchange factor, regulator of chromatin condensation 1 (RCC1) [19]. Unlike the bZIP transcription factor ELONGATED HYPOCOTYL 5 (HY5) and the COP1 E3 ubiquitin ligase, which are key regulatory factors in the UV-B-induced photomorphogenic pathway [12,19,20,21] and are involved in blue/UV-A and red/far-red light signaling pathways, UVR8 appears to be specific for the UV-B light response because *CHS* gene activation remains unaltered by red, far-red, or blue light or by non-light stimuli such as low temperature or sucrose in *uvr8* mutant plants [10,20].

UVR8 is a UV-B photoreceptor that employs specific tryptophans (Trps) in its primary sequence as chromophores in photoreception [18]. With the exception of UVR8, all photoreceptors contain specific external cofactors as chromophores: bilin for phytochrome, FAD and MTHF for cryptochrome, and FMN for phototropin. Tryptophan (Trp), which has an absorption maximum at approximately 280 nm (which extends to 300 nm and is probably shifted further in a protein environment), is particularly well suited to be a potential UV-B chromophore [22,23]. UVR8 has 14 highly conserved Trps, 1 in the C-terminus, 7 in the dimer interface, and 6 in the β-strands [18]. Two of these tryptophans, Trp 285 and Trp 233, collectively serve as the ultraviolet-B chromophore [24,25]. Recent studies of the crystal structure of UVR8 (amino acids 12–381) revealed how the dimer is maintained and how UV-B is sensed by specific Trps [24,25]. Besides, Arginine (Arg) residues, principally Arg 286 and Arg 338, play an important role in stabilizing the homodimeric interface by maintaining intermolecular hydrogen bonds with Asp or Glu residues from the neighboring UVR8 molecule [24]. Moreover, UV-B-dependent monomerization of the UVR8 homodimer occurs both *in vitro* and *in vivo* [6,24,25].

Although the signal transduction mechanism by which plant UVR8 functions as a UV-B photoreceptor is not fully understood, solid evidence indicates that COP1 and HY5, two common elements in light signaling, play major roles in promoting UV-B-induced photomorphogenesis. In plants, COP1 is a multifunctional protein best known for its role as a repressor of photomorphogenesis [26]. Following UV-B perception, UVR8 interacts with the WD40-repeat domain of COP1, and this interaction is closely linked to downstream UV-B-specific responses. Two separate domains of UVR8, the β-propeller domain and the C-terminal C27 domain, are both necessary and sufficient for interaction with COP1 [27,28]. The basic leucine-zipper transcription factor HY5 plays an important role in de-etiolation, the process by which plants adjust from growth in darkness to growth in light [29]. Both UVR8 and COP1 are required for the UV-B-mediated activation of *HY5* gene expression [20,21]. Nuclear-localized UVR8 was shown to associate with chromatin containing the promoter region of UV-B-responsive genes, such as *HY5* [30]. *HY5* is activated transcriptionally by UV-B in a UVR8- and COP1-dependent manner [10,12,20,21] and, in combination with the transcriptional activation of *HY5* expression, the HY5 protein is also stabilized by UV-B [10].

Although UV-B photoreceptors play important roles in the regulation of plant growth and development processes [10,19,20,31,32], most studies on UV-B photoreceptors have focused only on Arabidopsis leaving the UV-B photoreceptors of forest tree species relatively uncharacterized. Previous studies suggest that, besides Arabidopsis, UV-B light affects the growth and development of many crops and trees, such as sorghum [33], mazie [34] and pinaceae species [35]. Because of the depletion of the ozone layer and the enhanced UV-B radiation impinge on the earth year by year, knowing the structure and functions of the UV-B photoreceptor in other species is of great help for agriculture and forestry production. In this study, we isolated the *PeUVR8* gene encoding a UV-B light receptor from the desert species *Populus euphratica*, analyzed its structure and relationship with UVR8 proteins from other species, and substantiated the regulatory role of *PeUVR8* in plant gene expression, hypocotyl growth inhibition, and anthocyanin accumulation. Our results indicate that *PeUVR8* plays an important role in the regulation of growth and development of Euphrates poplar.

## Results

### Cloning of a Full-Length *PeUVR8* cDNA

To isolate a full-length cDNA sequence corresponding to the Euphrates poplar UV RESISTANCE LOCUS 8 (*PeUVR8*) gene, a Euphrates poplar expressed sequence tag (EST) clone with similarity to the Arabidopsis *UVR8* gene was identified by mining the NCBI EST database (dbEST) for Euphrates poplar ESTs isolated from Euphrates poplar leaves. Using a 504-bp EST fragment as a probe, we used the 5’/3’ rapid amplification of cDNA ends (5’/3’-RACE) extension method to obtain the missing *PeUVR8* RNA sequence. We obtained a 338-bp fragment corresponding to the sequence upstream of the EST sequence and a 720-bp fragment corresponding to the sequence downstream of the EST sequence. The EST sequence and the two fragments were then combined using DNAMAN software to generate the full-length *PeUVR8* sequence.

**Fig 1:**
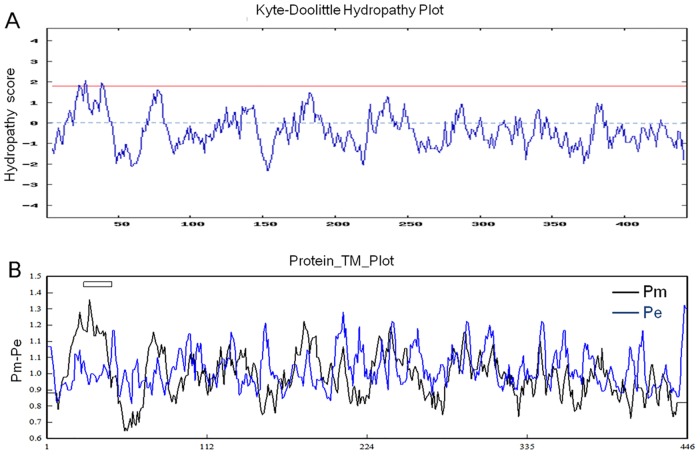
Hydrophilicity/hydrophobicity analysis and PeUVR8 transmembrane domain prediction. (A) Hydrophilicity/hydrophobicity analysis of PeUVR8 performed using the Kyte and Doolittle method. A score of 1.8 is indicated by the red line. (B) PeUVR8 transmembrane domain prediction using the DNAMAN version 5.2.2 software with default options. Predicted transmembrane regions are indicated by boxes above the profile.

We obtained a 1,563-bp, full-length *PeUVR8* cDNA sequence containing a 1,341-bp open reading frame (ORF) (GenBank accession number: KR052017). The *PeUVR8* ORF encoded a protein of 446 amino acids with a predicted mass of 47.83 kDa calculated using the DNAstar software. The deduced protein was basic with an isoelectric point (pI) of 5.92 predicted by the DNAMAN software. Hydrophilicity/hydrophobicity analysis of PeUVR8 using the Kyte and Doolittle method (http://gcat.davidson.edu/DGPB/kd/kyte-doolittle.htm) [36] showed that the majority of the PeUVR8 amino acids and almost all of the C-region amino acids were hydrophilic, indicating that PeUVR8 is a hydrophilic protein (Fig 1A). In addition, the Kyte and Doolittle method predicted a potential transmembrane region with a score greater than 1.8 (Fig 1A). We confirmed the results of the Kyte and Doolittle method by examining the transmembrane regions of PeUVR8 using DNAMAN software, which identified a potential transmembrane region between amino acids 22 and 50 (Fig 1B).

### Analysis of the PeUVR8 Amino Acid Sequence

**Fig 2:**
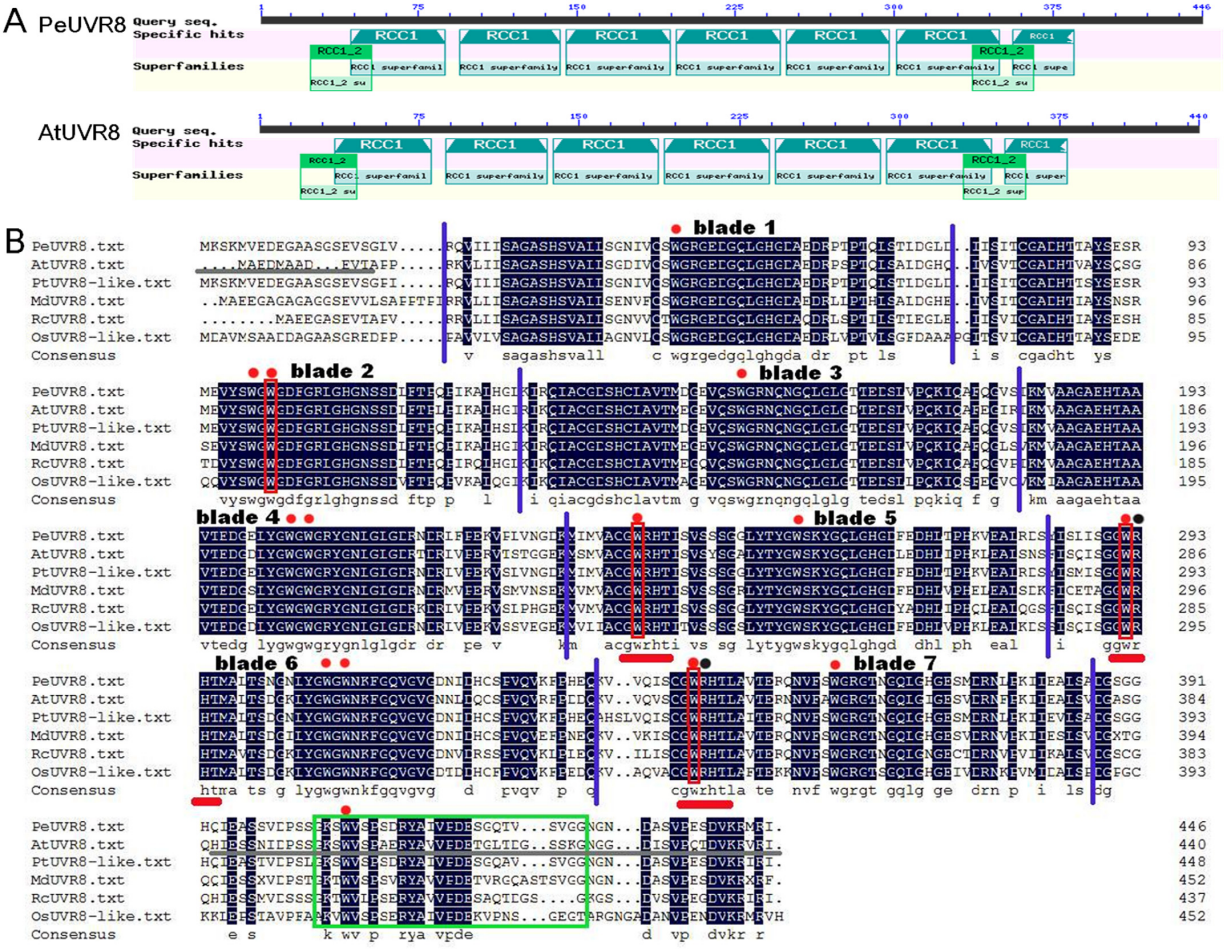
PeUVR8 protein sequence analysis. (A) Structural domains of the PeUVR8 and AtUVR8 proteins. Analysis of protein sequences in the National Center for Biotechnology Information (NCBI) database was performed using the CD-search software. (B) Amino acid sequence alignment of UVR8 proteins from Euphrates poplar, Arabidopsis, *Populus trichocarpa*, apple, castor, and rice. The alignment was constructed using the DNAMAN version 5.2.2 software. Identical residues are indicated by black boxes. Red dots above the sequences indicate the 14 conserved tryptophan residues and the red frames indicate the triad tryptophans (positions 233, 285, 337 in the Arabidopsis sequence) that form a pyramid arrangement with W94 on the adjacent monomer. Black dots above the sequences indicate the two arginine residues (positions 286, 338 in the Arabidopsis sequence) that are key to maintaining the AtUVR8 dimer. Red lines under the sequences indicate the conserved “GWRHT” motifs and the green box indicates the conserved C27 region. Purple lines indicate the seven blades compared with AtUVR8. Grey lines under the AtUVR8 sequence indicate the missing residues in the AtUVR8 crystal structure.

UVR8 proteins occur widely among plant species and are well conserved [6]. Sequence analysis of the NCBI Conserved Domain Database using the CD-search tool (http://structure.ncbi.nlm.nih.gov/Structure/cdd/wrpsb.cgi) revealed that, like the AtUVR8 protein, the PeUVR8 amino acid sequence contained nine well-conserved RCC1 repeats belonging to seven RCC1 superfamilies and two RCC1_2 superfamilies, (Fig 2A).

In Arabidopsis, the UVR8 core domain forms a seven-bladed β-propeller. Similar to AtUVR8, PeUVR8 consists of a seven-bladed β-propeller fold with a short N-terminal extension and a flexible C-terminal region (Fig 2B). Besides, AtUVR8 has 14 highly conserved Trps, all of which are conserved in PeUVR8 (Fig 2B). Moreover, the clustered triad Trps, W233, W285 and W337, which form a pyramidal arrangement with W94 on the adjacent monomer, the two Trps, W233 and W285, which collectively serve as the ultraviolet-B chromophore, and the two Args, R286 and R338, which are key to maintaining the dimer [24,25], were well conserved in PeUVR8 (Fig 2B).

In Arabidopsis, the triad of closely packed tryptophans—W233, W285, and W337—that are implicated in UVR8 photoreception [6], is generated by a conserved Gly-Trp-Arg-His-Thr sequence repeat (GWRHT) in blades 5, 6, and 7 [24,25]. In PeUVR8, all of the three GWRHT motifs were well conserved, and also distributed in corresponding blades (Fig 2B). Additionally, the C27 region, which contains stretches of amino acids that are highly conserved in UVR8 sequences from various plant species and crucial for interaction with COP1 [27], was also present and conserved in the PeUVR8 C-terminal region (Fig 2B).

### Structural Analysis of the PeUVR8 Amino Acid Sequence

**Fig 3:**
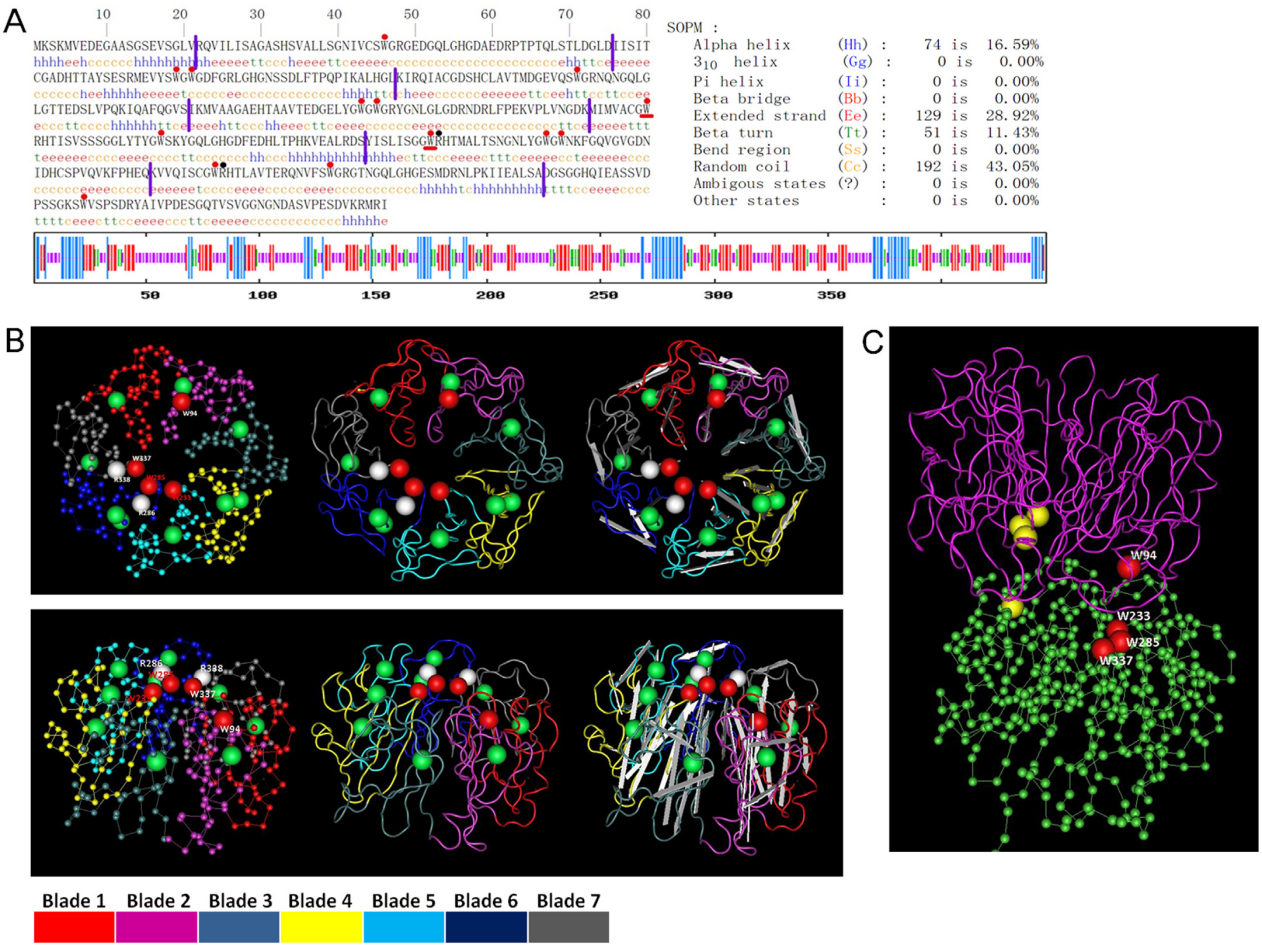
Structural analysis of the PeUVR8 protein. (A) The secondary structure of PeUVR8 solved using the self-optimized prediction method (SOPM). Red dots above the sequence indicate the 14 conserved tryptophans. Red lines and black dots indicate the crucial two tryptophans (W233, W285) and two arginines (R286, R338) corresponding to AtUVR8, respectively. Purple lines indicate the seven blades of PeUVR8 compared with AtUVR8. (B) Comparison of the predicted three-dimensional structures of PeUVR8 and AtUVR8 using Cn3D software. Views from the top (above image) and from the side (image below). Red and green balls in the images indicate the conserved tryptophans with the red balls indicating the four crucial tryptophans (W94 and the clustered triad tryptophans W233, W285, W337) used to form pyramids between the UVR8 dimer. White balls indicate the two arginines (R286, R338) that are crucial for maintaining the dimer structure of UVR8. The seven blades of UVR8 are represented by different colors (under the two images). (C) 3D structure of the UVR8 dimer (viewed from the side). Red and yellow balls indicate the two pyramids between the two monomers.

The secondary structure of PeUVR8 was solved using the self-optimized prediction method (SOPM, http://npsa-pbil.ibcp.fr/cgi-bin/npsa_automat.pl?page=npsa_sopm.html) [37]. The PeUVR8 protein contained α-helix (74 aa, 16.59%), β-turn (51 aa, 11.43%), extended strand (129 aa, 28.92%), and random coil (192 aa, 43.05%) regions (Fig 3A). The α-helices and β-turns were distributed throughout the PeUVR8 polypeptide. Comparison of the PeUVR8 with AtUVR8 structures using the Cn3D macromolecular structure viewer (http://www.ncbi.nlm.nih.gov/Structure/CN3D/cn3d.shtml) showed that these two proteins have similar structures. Using the crystal structure of the AtUVR8 core domain (PDB: 4NWD_A; lacking 11 amino acids at the N-terminus and 59 amino acids at the C-terminus) as a model, the corresponding domain of PeUVR8 forms a seven-bladed β-propeller (Fig 3B). Moreover, among the 14 conserved Trps, with the exception of one missing in the C-terminus, seven are in the dimer interface and six in the β-strands (Fig 3B), similar to AtUVR8 [24,25]. The two pyramids per dimer, which are formed by the triad Trps (W233, W285, W337) on one monomer and the W94 on another monomer, are present and conserved (Fig 3B and 3C).

**Fig 4:**
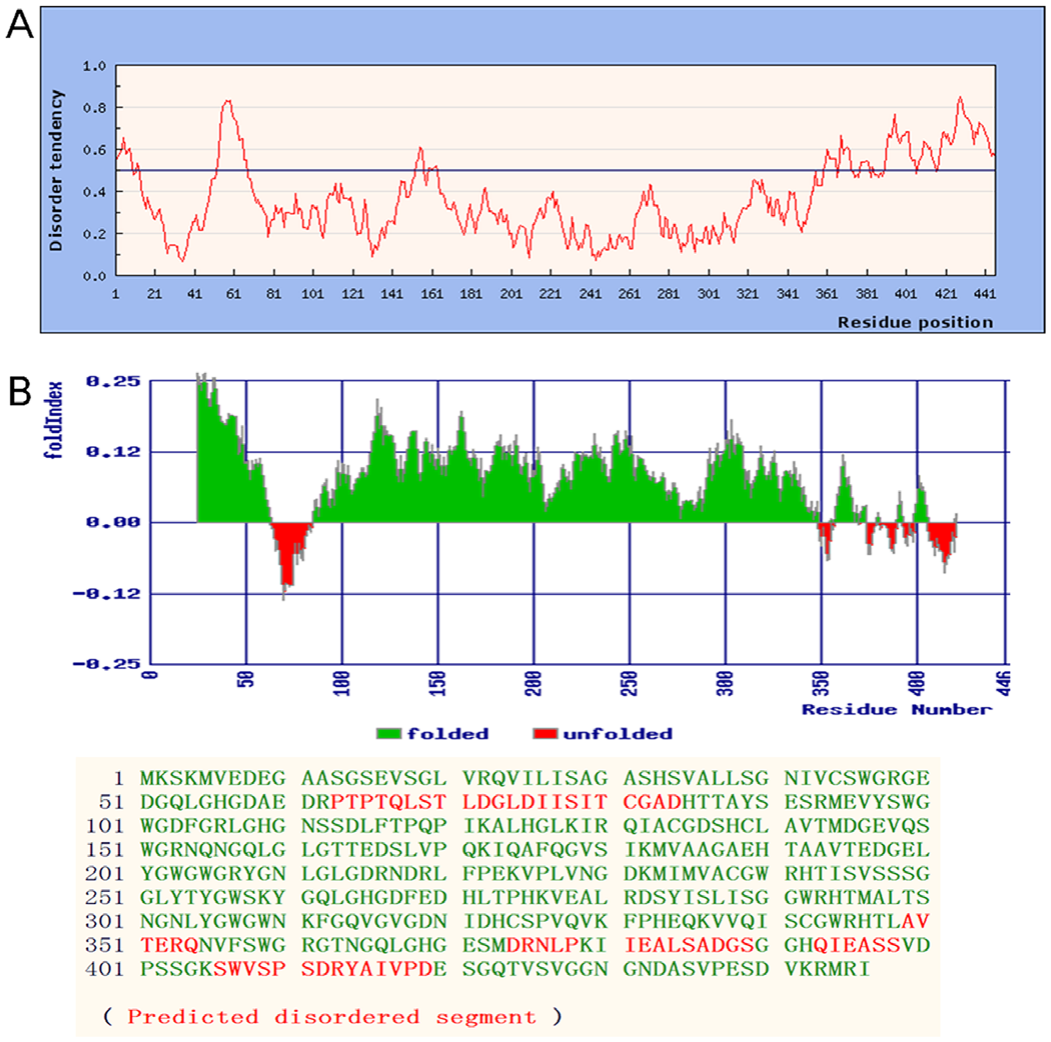
Local disorder tendency prediction and fold disordering character analysis of PeUVR8 protein. (A) Local disorder tendency of PeUVR8 based on an estimated-amino-acid-pairwise-energy-content analysis using the IUPred software. (B) The fold disordering character of PeUVR8 predicted using the FoldIndex software.

It was reported that the UVR8 crystal structures do not contain the C-terminal region (59 amino acids) because the presence of the C-terminal region prevents crystallization of UVR8, suggesting that the C-terminal region is flexible [24,25,27]. For a more detailed understanding of the PeUVR8 structure, we investigated the local disorder tendency of the PeUVR8 amino acid sequence based on an estimated-amino-acid-pairwise-energy-content analysis using the IUPred web server (http://iupred.enzim.hu/), which showed that the C-terminal region of PeUVR8 is intrinsically unstructured, especially in the last 60 amino acids (386aa-446aa) (Fig 4A). To confirm this result, we examined the fold disordering character of PeUVR8 using the FoldIndex software (http://bip.weizmann.ac.il/fldbin/findex) [38], and the result was in agreement with that obtained using the IUPred web server (Fig 4B).

### Relationship of PeUVR8 to Other Plant UV-B Receptors

**Fig 5:**
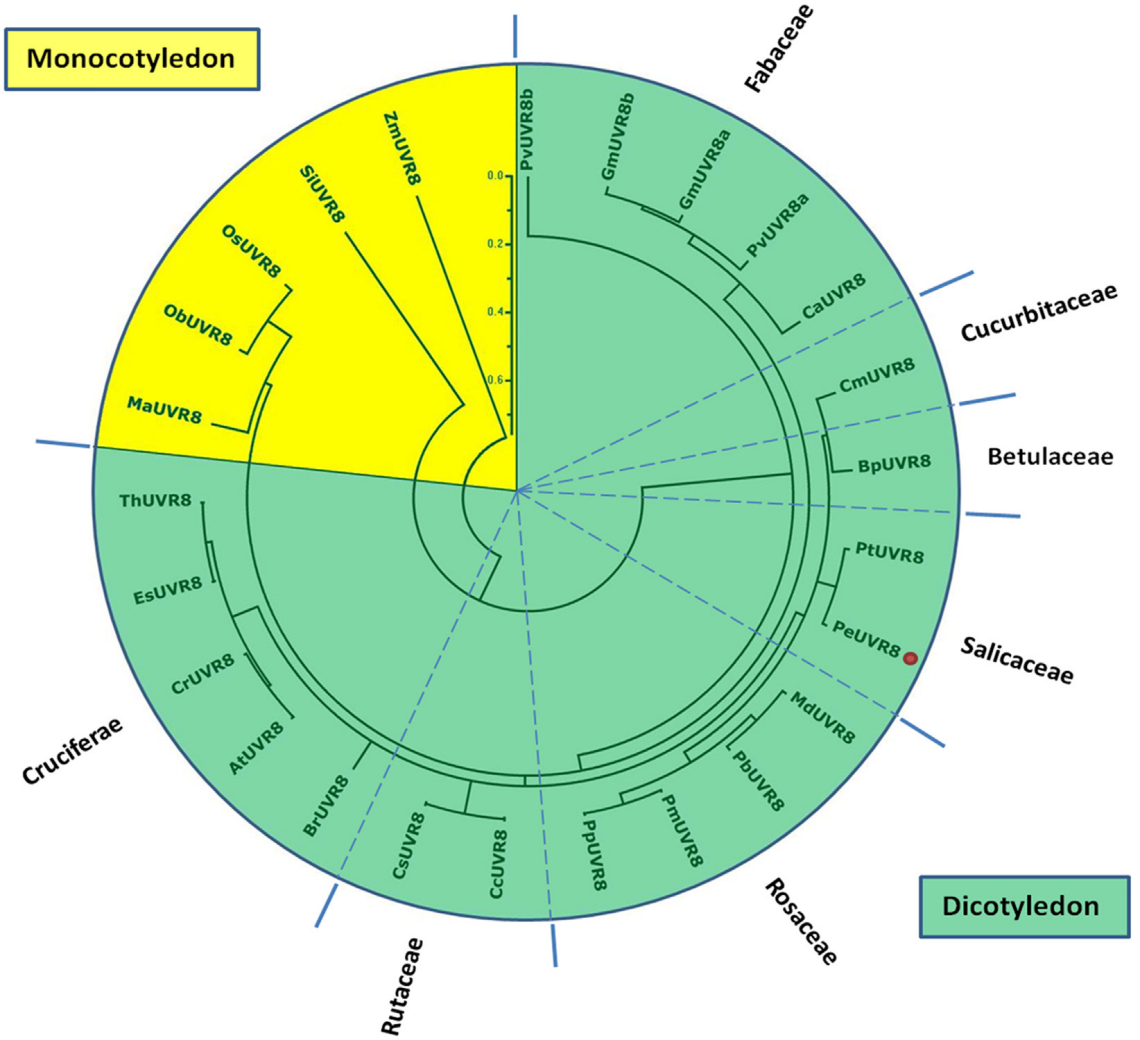
Phylogram of plant UVR8 proteins. UVR8 amino acid sequences from 22 diverse plant species were obtained from the NCBI database. The alignment was constructed using ClustalX and the phylogenetic tree was constructed using the neighbor-joining method in the MEGA version 4.1 software. Each node corresponds to a number indicating the bootstrap value for 1000 replicates. The scale bar represents 0.1 substitutions per sequence position. PeUVR8 is denoted by a red dot. *Pv*, *Phaseolus vulgaris*; *Gm*, *Glycine max*; *Ca*, *Cicer arietinum*; *Cm*, *Cucumis melo*; *Bp*, *Betula platyphylla*; *Pt*, *Populus trichocarpa*; *Pe*, *Populus euphratica*; *Md*, *Malus domestica*; *Pb*, *Pyrus bretschneideri*; *Pm*, *Prunus mume*; *Pp*, *Prunus persica*; *Cc*, *Citrus clementina*; *Cs*, *Citrus sinensis*; *Br*, *Brassica rapa*; *At*, *Arabidopsis thaliana*; *Cr*, *Capsella rubella*; *Es*, *Eutrema salsugineum*; *Th*, *Thellungiella halophila*; *Ma*, *Musa acuminate*; *Ob*, *Oryza brachyantha*; *Os*, *Oryza sativa*; *Si*, *Setaria italic*; *Zm*, *Zea mays*.

To analyze the phylogenetic relationship between PeUVR8 and UVR8 proteins from other plant species, we performed phylogenetic analysis of 25 plant UVR8 proteins representing 23 diverse species, including 18 dicotyledonous species distributed among 7 genera, using the Mega 4.1 software and the Clustal method (Fig 5). The phylogenetic tree revealed a clear boundary between the UVR8 proteins of dicotyledonous and monocotyledonous plants. PeUVR8 grouped in the dicotyledon UVR8 clade and was most closely related to the *Populus trichocarpa* UVR8 protein PtUVR8 (XM_002309903), which clustered in the same clade. In contrast, PeUVR8 was most distantly related to the UVR8 proteins of the monocotyledonous species including banana MaUVR8 (XM_009406583), rice OsUVR8 (NM_001059384) and ObUVR8 (XM_006652246), millet SiUVR8 (XM_004969748), and maize ZmUVR8 (XM_008658845).

### Analysis of Tissue-Specific *PeUVR8* Expression

**Fig 6:**
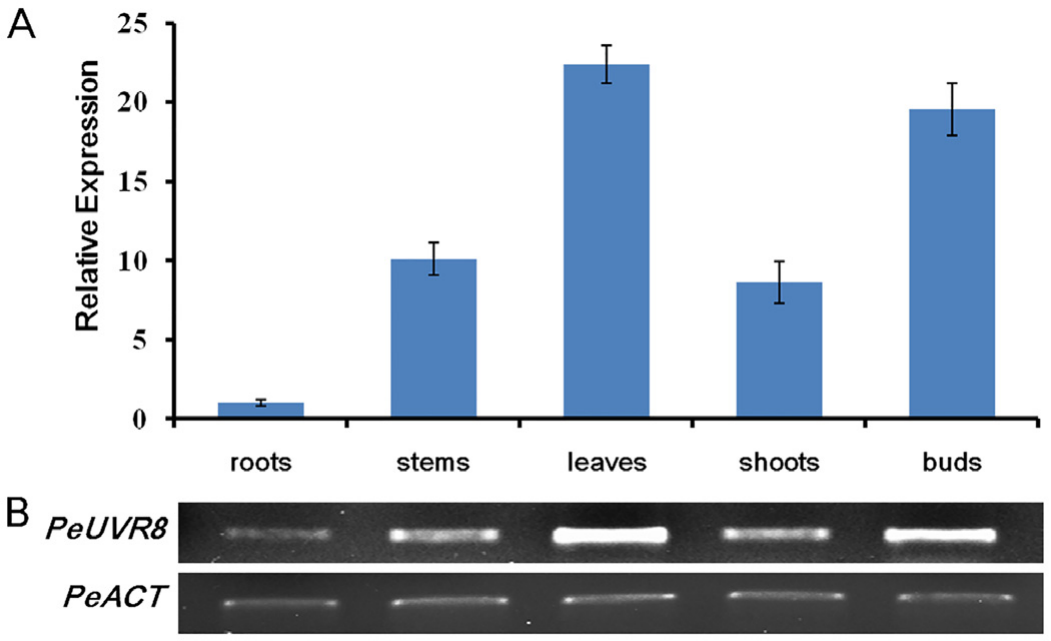
Analysis of tissue-specific *PeUVR8* expression. Analysis of *PeUVR8* expression in various tissues (roots, stems, leaves, shoots and buds) using (A) real-time quantitative and (B) semiquantitative RT-PCR. The *PeACT* gene was performed as an internal control. Error bars indicate s.e. over three biological replicates (each replicate, three technical replicates).

Previous studies showed that *AtUVR8* was expressed ubiquitously in all cell types and organs examined [10], and that the constitutive expression of UVR8 allows any plant organ to respond immediately to UV-B exposure and to mount protective responses [6]. To determine whether *PeUVR8* transcript levels were tissue-specific, we performed semiquantitative and real-time quantitative reverse transcription (qRT)-PCR analysis using total RNAs obtained from various tissues, including roots, stems, shoots, buds, and leaves. *PeUVR8* was expressed in all tissues examined, but the expression levels varied among the tissues (Fig 6A and 6B). *PeUVR8* expression was highest in the leaves and buds, followed by the shoots and stems, with relatively weak expression detected in the roots.

### Functional Complementation Assay of *PeUVR8* in an *Arabidopsis uvr8* Mutant

**Fig 7:**
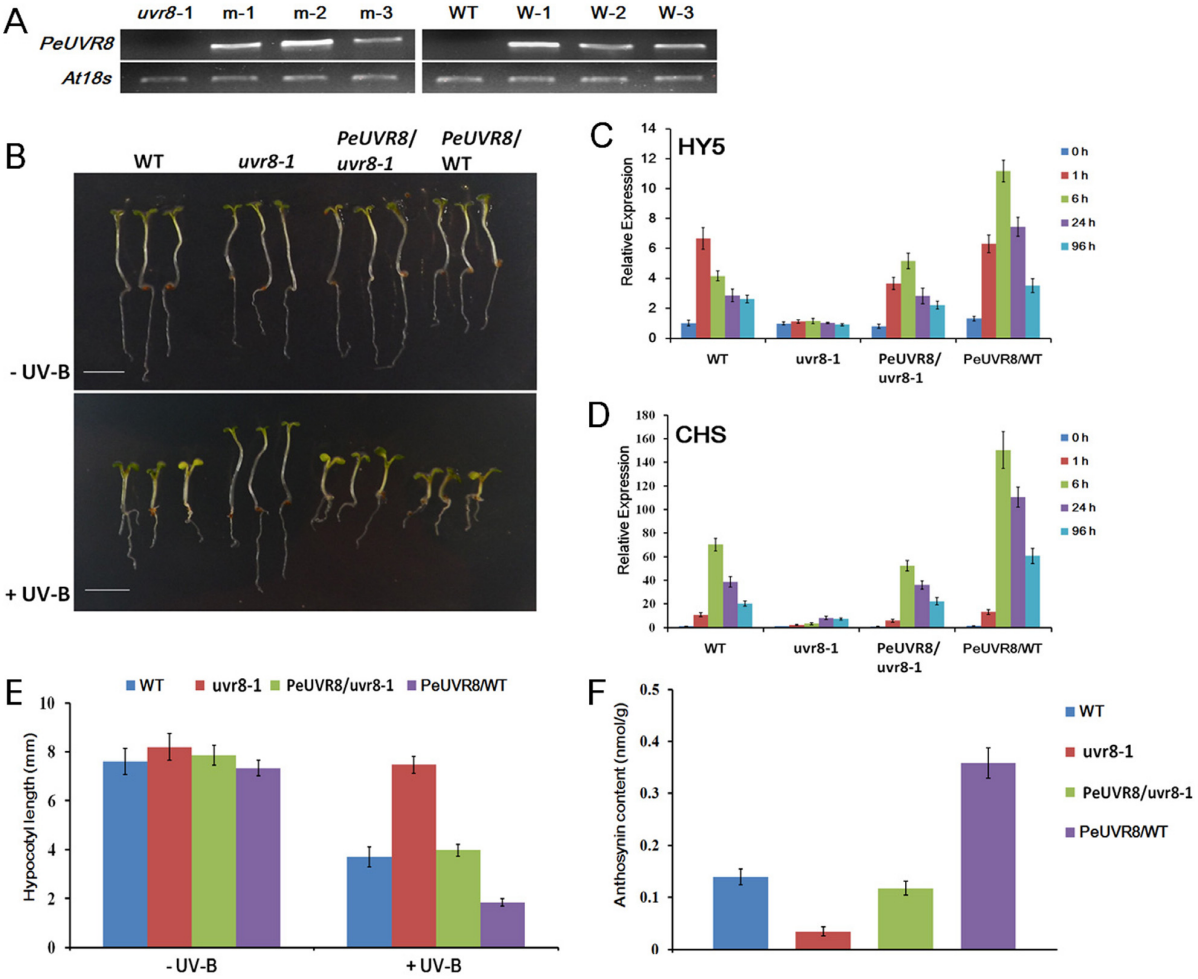
Phenotypes of wild-type (WT), *uvr8-1* mutant, *PeUVR8*-transgenic *uvr8-1* mutant, and *PeUVR8*-transgenic WT plants. (A) *PeUVR8* transcript levels in 4-day-old seedlings of WT, *uvr8-1* mutant, and transgenic lines. Three *uvr8-1* mutant lines transformed with *PeUVR8* are designated m-1, m-2, and m-3. Three WT lines transformed with *PeUVR8* are designated W-1, W-2, and W-3. (B) Phenotypes of the WT, *uvr8-1* mutant, and two transgenic lines (m-2 and W-1) grown under white light with or without narrowband UV-B light. Scale bar represents 5 mm. (C) and (D), Quantitative RT-PCR analysis of *HY5* and *CHS* gene expression levels in the WT, *uvr8-1* mutant, and the two transgenic lines with different durations of UV-B irradiation. Error bars indicates s.e. over three biological replicates (each replicate, 10–15 pooled seedlings). (E) Hypocotyl lengths of 4-day-old seedlings of WT, *uvr8-1* mutant, and two transgenic lines grown under white light with or without narrowband UV-B light. Error bars indicates s.d. (n > 30). (F) Anthocyanin content of 4-day-old seedlings of WT, *uvr8-1* mutant, and two transgenic lines grown under white light with narrowband UV-B light. Error bars indicates s.d. (n > 30).

In Arabidopsis, overexpression of *UVR8* resulted in an enhanced UV-B photomorphogenic response that included activation of HY5 and CHS gene expression, hypocotyl growth inhibition, and anthocyanin accumulation [10]. To investigate the function of *PeUVR8* in plants, we conducted a functional complementation assay using an Arabidopsis *uvr8-1* mutant. *PeUVR8* was introduced into the pRI 101-AN vector with expression driven by a cauliflower mosaic virus (CaMV)-35S promoter followed by a 58-bp Arabidopsis alcohol dehydrogenase (*AtADH*) 5’UTR enhancer. The plasmid carrying the 35S::PeUVR8 expression cassette was transformed into wild-type (WT; Landsberg *erecta*) and *uvr8-1* mutant Arabidopsis backgrounds using the floral dip method [39]. After repeated selection on kanamycin and PCR screening for the presence of the transgene, at least three transformants of each background were obtained. Transgene expression levels were determined by semiquantitative RT-PCR to verify successful transformation (Fig 7A). Two transgenic lines, m-2 and W-1, with high levels of *PeUVR8* expression were chosen to compare the phenotypes of WT, *uvr8-1* mutant, *uvr8-1* mutant transformed with *PeUVR8*, and *PeUVR8* overexpressing lines.

#### *PeUVR8* Promotes Gene Expression of *CHS* and *HY5* in *Arabidopsis*

A previous study showed that the *uvr8* mutant in Arabidopsis, unlike a variety of UV-B-hypersensitive mutants that are defective in DNA damage repair or sunscreen biosynthetic enzymes [19], exhibited altered UV-B signal transduction as indicated by a lack of UV-induced flavonoid accumulation and *CHS* and *HY5* gene expression [10]. To determine if *PeUVR8* promoted *CHS* and *HY5* expression in Arabidopsis under UV-B irradiation, 4-day-old seedlings of the four types of Arabidopsis lines were exposed to white light with supplemental UV-B light for periods of 0–96 h and the transcript levels of these two genes were determined by real-time qRT-PCR analysis. Under irradiation with white light (0 h), both the *CHS* and *HY5* genes exhibited low basal expression levels in the four lines (Fig 7C and 7D), but under UV-B irradiation, the *uvr8* mutant seedlings exhibited a lack of UV-induced increases in *CHS* and *HY5* expression. Transformation of the *uvr8-1* mutant with *PeUVR8* restored the UV-induced increases in *CHS* and *HY5* expression. Moreover, the *PeUVR8*-overexpressing lines exhibited the highest *CHS* and *HY5* transcript levels of the three genotypes (Fig 7C and 7D) as was observed previously in Arabidopsis lines overexpressing *AtUVR8* [10]. Although *CHS* and *HY5* function downstream of UVR8 protein in the UV-B signal transduction mechanism, the expression levels of both genes rapidly reached a peak within 6 h in both the WT and the *PeUVR8*-overexpressing lines (Fig 7C and 7D) which was consistent with the rapid monomerization of UVR8 after UV-B treatment [6]. These results demonstrated that *PeUVR8* played the same role as *AtUVR8* in the promotion of *CHS* and *HY5* gene expression in plants.

#### *PeUVR8* Inhibits Hypocotyl Elongation in *Arabidopsis*

Hypocotyl length is the phenotype most commonly used to study the functions of photoreceptors in Arabidopsis. To increase our understanding of the role of *PeUVR8* in regulating UV-B-induced photomorphogenesis, we examined UV-B-responsive hypocotyl growth inhibition in the four types of Arabidopsis lines. When the seedlings were grown under white light, the hypocotyl lengths did not differ significantly among the four genotypes (Fig 7B and 7E). However, when grown under white light supplemented with narrowband UV-B light, the *uvr8-1* mutant lines exhibited reduced inhibition of hypocotyl elongation compared with WT and transformation of the *uvr8-1* mutant with PeUVR8 partially restored the WT phenotype. In addition, the *PeUVR8*-overexpressing lines exhibited the shortest hypocotyl lengths of the three genotypes (Fig 7B and 7E). These results demonstrated that *PeUVR8* inhibited hypocotyl elongation in plants under specific UV-B conditions, similar to *AtUVR8*.

#### *PeUVR8* Increases Anthocyanin Accumulation in *Arabidopsis*

Previous studies showed that, under specific UV-B irradiation conditions, an approximately 50% inhibition of hypocotyl growth was accompanied by anthocyanin and flavonoid accumulation in Arabidopsis [21]. In addition, anthocyanin accumulation was shown to decrease markedly in the Arabidopsis *uvr8* mutant and increase significantly in Arabidopsis seedlings overexpressing *AtUVR8* grown under white light supplemented with narrowband UV-B light [10]. In this study, transformation with *PeUVR8* also resulted in significantly increased anthocyanin accumulation in both the *uvr8-1* mutant and WT seedlings under specific UV-B irradiation conditions (Fig 7F). These results suggest that *PeUVR8* promotes anthocyanin accumulation under specific UV-B conditions.

### PeUVR8 Interacts with AtCOP1 *in vivo*

Although a recent study identified UVR8 as the long-sought-after UV-B photoreceptor [6], the signal transduction mechanism of this type of photoreceptor in plants is not fully understood. Recent studies suggested that the interaction between UVR8 and COP1 play a crucial role in UV-B perception and is closely linked to downstream UV-B specific responses [27,28]. Because of the highly similar structures and functions of PeUVR8 and AtUVR8, we performed bimolecular fluorescence complementation (BiFC) assays to determine whether PeUVR8 interacts with AtCOP1 and whether PeUVR8 function is conserved between poplar and Arabidopsis.

**Fig 8:**
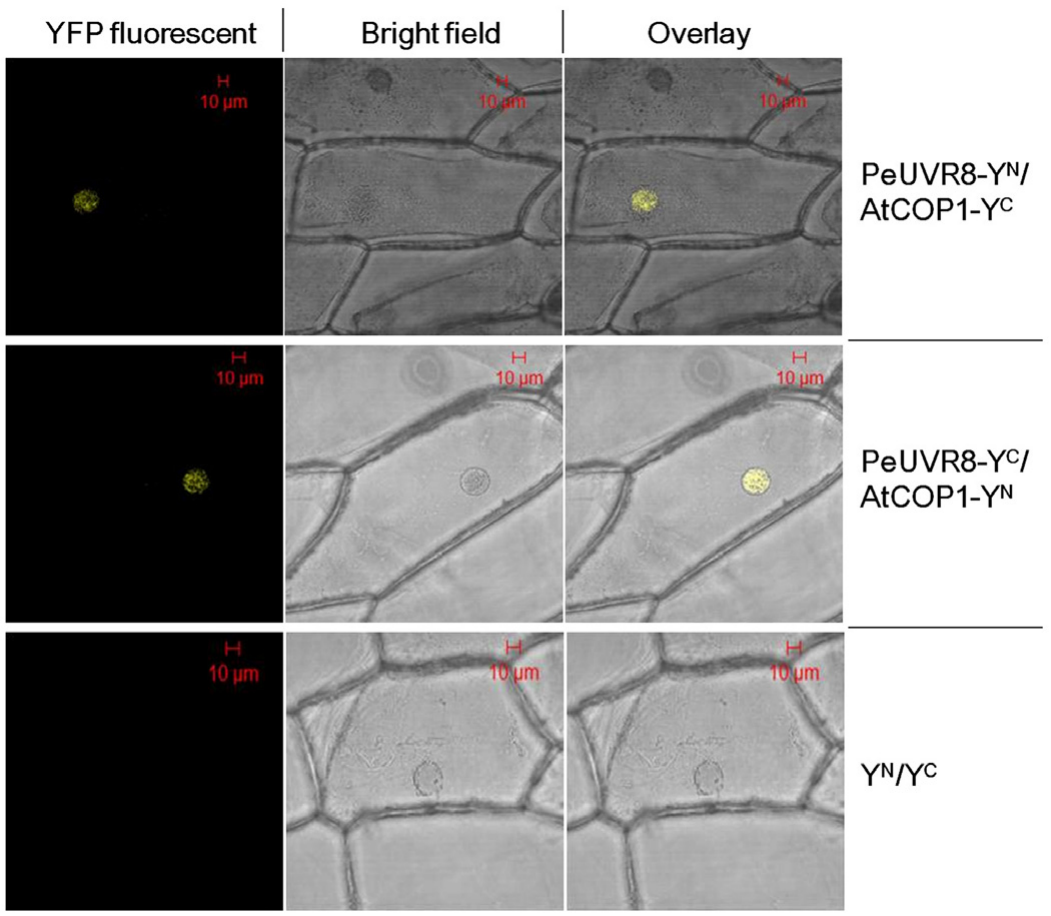
PeUVR8 interaction with AtCOP1 in bimolecular fluorescence complementation (BiFC) assays. The right images are the overlay of YFP fluorescent (left) and bright-field (middle) images of onion epidermal cells cotransformed with indicated combinations. Y^N^ and Y^C^ represent the N- and C-terminal regions of YFP respectively.

Full-length *PeUVR8* and *AtCOP1* cDNAs were fused to the N-terminal (Y^N^) and C-terminal (Y^C^) halves, respectively, of yellow fluorescent protein (YFP), driven by a cauliflower mosaic virus (CaMV)-35S promoter. The fusion proteins were then introduced transiently into onion epidermal cells with different combinations (Fig 8). Under specific UV-B irradiation conditions (1.5 μmol m^–2^ s^–1^ narrowband UV-B), YFP fluorescence was predominantly detected in the nuclei of the cells cotransformed with two combinations: PeUVR8-Y^N^ plus AtCOP1-Y^C^ and PeUVR8-Y^C^ plus AtCOP1-Y^N^. In contrast, no fluorescence was observed in the control combination including the two empty vectors: pSPYNE-35S (Y^N^) plus pSPYCE-35S (Y^C^). These results suggested that PeUVR8 and AtCOP1 interact *in vivo* in plant cells (Fig 8), and provided additional evidence that PeUVR8 is the poplar counterpart of AtUVR8 and plays a role in the molecular mechanism of the UV-B light signal transduction pathway in poplar.

## Discussion

Plants are unavoidably exposed to UV-B radiation in sunlight due to their sessile lifestyle and their need to capture light to drive photosynthesis. UV-B light is a key environmental signal that is specifically perceived by plants to promote UV acclimation and survival in sunlight. Whereas the plant photoreceptors for visible light are rather well characterized, the UV-B photoreceptor UVR8 was only recently described at the molecular level [29]. Since the discovery of this gene [19], studies over the past 10 years have shown that UVR8 proteins occur widely in plants and are well conserved. In this study, we cloned the *PeUVR8* gene from Euphrates poplar and demonstrated its function as a UV-B receptor similar to that of *AtUVR8* in Arabidopsis.

Recent studies of UVR8 crystal structures indicated that there are 14 Trps, 1 in the C-terminus, 7 in the dimer interface, and 6 in the β-strands (Fig 3B). The six Trps in the β-strands were shown to help maintain the propeller structure, as they form hydrogen bonds and hydrophobic interactions between adjacent blades [40]. Two Trps, W233 and W285, which are the principal Trps required for UV-B photoreception, are not functionally redundant and evidently have distinct roles in the UVR8 mechanism. Mutation analysis of these two Trps indicated these important roles in UV-B perception and conformational change of UVR8, but the present results suggest a prior role for W285 in UVR8 photoreception [24,25]. It was reported that the variant W285A appeared only as a monomer both before and after ultraviolet-B irradiation and, in contrast, the variant W285F was unable to undergo ultraviolet-B-induced monomerization [24]. Remarkably, although the UVR8 variant W285F is unable to respond to UV-B, it does respond to UV-C, consistent with the shorter wavelength absorption of phenylalanine compared with tryptophan [25]. These results not only demonstrate the key role of W285 as the principal UV-B sensor, but also show that the spectral sensing properties of the photoreceptor can be retuned by a single amino acid change. W233 is also important and the variant W233F abrogated dimerization of UVR8 monomers [6], and exhibited the greatest reduction in far-UV CD peak height among the single conservatively substituted W>F mutants (W233F, W285F, W337F) [25].

Although the crystal structures of UVR8 and the chromophore Trps have been identified, little is known about how UV-B photoreception leads to monomerization of the protein. There are two main hypotheses, one emphasizes the important roles of cation-Pi interactions between the triad Trps and adjacent salt-bridging Args, which could be weakened by UV-B photoreception [24], and the other suggests that excitation of excitonically coupled triad Trps could result in the effective transfer of an electron to adjacent Arg residues, causing neutralization of salt bridges. These hypotheses still need to be confirmed experimentally, but solid data show the crucial role of the triad Trps (W233, W285, W337) and the two Args (R286, R338) in maintaining the dimer structure of UVR8 and in UV-B photoresponse [24,25]. Using the crystal structure of AtUVR8 as a model, comparison of the structures of PeUVR8 and AtUVR8 showed that these two proteins could have similar spatial structures (Fig 3B). The presence of the conserved residues and domains and the similar spatial structure indicated that PeUVR8 function should be similar to that of AtUVR8.

Analysis of phylogenetic relationship between PeUVR8 and UVR8 proteins from other plant species suggested that PeUVR8 was most closely related to the *Populus trichocarpa* UVR8 protein PtUVR8, which is consistent with the fact that these two plants belong to the same genus. However, in the phylogenetic tree, it seems that ZmUVR8 and OsUVR8 are the most distant UVR8 homologues. Because the UV-B photoreceptor *UVR8* was identified recently [6] and most studies on UV-B photoreceptors have focused only on Arabidopsis, most of the sequences of UV-B photoreceptors in other species were unknown. In this study, some of the amino acid sequences we downloaded from NCBI and used to construct the Phylogenetic tree are just sequences of predicted ultraviolet-B receptor UVR8-like genes, such as ObUVR8 (XM_006652246), millet SiUVR8 (XM_004969748), and maize ZmUVR8 (XM_008658845). These sequences may not be the exact UVR8 amino acid sequences in these species, resulting the distance between ZmUVR8 and OsUVR8.

Based on the high similarity of the predicted secondary and tertiary structures of PeUVR8 and AtUVR8, we propose that PeUVR8 may be a UV-B light photoreceptor in Euphrates poplar. Moreover, we come up with a hypothesis that, like AtUVR8, PeUVR8 undergoes a UV-B-induced conformational change to transmit the light signal through interaction with other proteins such as COP1 to regulate plant growth and development [6,10]. The results of the BiFC assay showing that PeUVR8 can interact with AtCOP1 under specific UV-B conditions (Fig 8) support this hypothesis and raise the possibility that one or more proteins like COP1 may interact with PeUVR8 in Euphrates poplar to regulate plant growth under UV-B irradiation. The identification of other proteins that can interact with PeUVR8 and how such protein-protein interactions might transmit UV-B signals await further study.

Analysis of *PeUVR8* expression in various Euphrates poplar tissues showed that *PeUVR8* was expressed in Euphrates poplar root tissue, although at a relatively low level (Fig 6). Given that the function of UVR8 is dependent on UV-B-induced monomerization of UVR8 dimers, how such a UVR8 UV-B photoreceptor can be activated in underground tissues without direct UV-B light is an interesting question. The blue light receptor cryptochrome can regulate the synthesis, transport, or concentration of hormones such as auxin [41,42,43], gibberellic acid (GA) [44], and ethylene [45]. The functions of both AtCRY1 and AtCRY2 in regulating primary root elongation have been shown to be dependent on the transport of auxin from the aerial part of the plant to the root tissues [46]. In addition, the red/far-red photoreceptor proteins PHYA and PHYB have been reported to be involved in root tissues in the transmission of some hormone signals from the shoot [47]. It is not known, with the exception of UV-B, if UVR8 function can also be activated by other signals as in other photoreceptors [46,48]. Other recent studies have hypothesized a root-specific UV-B response that may represent a novel UV-B sensing mechanism [49]. This proposed root-specific pathway, however, is linked to vitamin B6 homeostasis and is clearly distinct from the UVR8/COP1-mediated signaling responsible for UV-B-induced photomorphogenesis and UV-B acclimation [50]. All of these hypotheses require further experimentation to be substantiated.

The results of the functional complementation assay of *PeUVR8* in *uvr8-1* mutant background—coupled with the protein interaction with AtCOP1 (Fig 8)—indicate that PeUVR8 is a counterpart of AtUVR8. The perception of UV-B by UVR8 followed by a UVR8-COP1 interaction has emerged as a primary mechanism of the UV-B response that is crucial for UV-B acclimation and tolerance [29]. That COP1 interacts with blue light, red light, and UV-B photoreceptors, but with different molecular outcomes of each interaction is remarkable. For example, COP1 mediates ubiquitination leading to degradation of the light-labile photoreceptors CRY2 and phyA, but it does not appear to affect the stability of other photoreceptors, such as phyB, CRY1, or UVR8 [10,26]. The fact that COP1 and HY5 are major downstream effectors in UV-B signaling as well as in visible light signaling indicates a high potential for cross talk between the UV-B and visible light responses. For example, part of the interaction of UVR8 with COP1 under extended UV-B irradiation might include removing COP1 from phytochrome and/or cryptochrome signaling [10]. The positive results of the PeUVR8-AtCOP1 interaction suggest that the UV-B perception and transduction mechanism in Euphrates poplar may identical to that in Arabidopsis. Additional studies are needed to fully clarify the correlation between UV-B and visible light signal transduction mechanisms and a detailed molecular map of UV-B and visible light signaling is essential to fully comprehend the regulatory role of UV-B and the control that sunlight exerts over plant growth and development.

## Materials and Methods

### Ethics statement

No specific permits were required for the described field studies. The location is not privately owned or protected in any way, and the field studies did not involve endangered or protected species.

### Plant material and growth conditions

Samples of roots, stems, shoots, buds, and leaves were collected from adult (about 50-year-old) *Populus euphratica* (Euphrates poplar) trees grown in natural conditions in Xinjiang province, China. The collected stem samples, which were about 1meter above the ground, mainly include the phloem, vascular cambium and small amount of xylem. And the root samples consist of lateral roots with diameter less than 4 mm. All of these samples were collected in March, 2013. The poplar forest was located along the Tarim River in western China (41.0526° N 86.2289° E).

An *Arabidopsis thaliana* ecotype Landsberg *erecta* (L*er*) line was used as the wild type (WT). Seeds of L*er*, *uvr8-1* mutant, and transgenic lines were sown on MS medium, cold-treated for 3 days at 4°C, and transferred to controlled environment cabinets under long-day (16-h light/8-h dark) conditions at 22°C.

Experiments involving UV-B light treatments were performed in a controlled environment chamber. Plants were grown under continuous low-fluence-rate white light (20 μmol m^–2^ s^–1^) supplemented with Philips TL20W/01RS narrowband UV-B tubes (1.5 μmol m^–2^ s^–1^). The UV-B range was modulated using 3-mm transmission cutoff filters of the WG series with half-maximal transmission at the indicated wavelength (WG305 and WG345). Seedlings were grown under continuous light supplemented with UV-B under a 345-nm cutoff filter (–UV-B) or 305-nm cutoff filter (+ UV-B).

### Isolation of full-length *PeUVR8* cDNA using rapid amplification of cDNA ends (RACE)

A 504-bp expressed sequence tag (EST) sequence encoding *PeUVR8* was isolated by reverse transcription-polymerase chain reaction (RT-PCR) using the degenerate primer pair U8EST-F (5’-AGTGATTTGTTCACTCCTCAG-3’) and U8EST-R (5’-CAAGGCTTCCACCTTGTGAGG-3’). We used 5’- and 3’-RACE to obtain the full-length gene. Total RNA was isolated from Euphrates poplar leaves using the TRIzol (Invitrogen, Carlsbad, CA, USA) method according to the manufacturer’s instructions. PCR products of the expected sizes were purified, cloned into the pMD18-T vector (Takara Bio, Otsu, Japan), and sequenced. The putative 3’- and 5’-RACE cDNA sequences were overlapped with the EST sequence using DNAMAN software to form a cDNA contig, which was used to determine the putative translation initiation codon (ATG) and open reading frame (ORF). To obtain a full-length *PeUVR8* cDNA, a pair of full-length primers PeUVR8-F (5’-GATATGAAAAGCAAAATGGTCG-3’) and PeUVR8-R (5’-GAATTATCAAATCCGCATCCG-3’) was designed based on the contig. The full length *PeUVR8* sequence was then obtained by RT-PCR using the full-length primers.

### Expression analysis

Plant materials were harvested, frozen in liquid nitrogen, and then ground under RNase-free conditions. RNA was extracted using TRIzol Reagent (Invitrogen), and treated with DNase I (Takara Bio) at 37°C for 30 min, following the manufacturer ‘s instructions. Then the RNA was reverse transcribed using the PrimeScript First Strand cDNA Synthesis Kit (Takara Bio) following the manufacturer’s instructions. A 10-μl aliquot of cDNA was diluted to a final volume of 100 μl with water.

Semiquantitative RT-PCR was carried out in 25-μl reactions with 5 ng of diluted cDNA template. The PCR profile was 94°C for 5 min, 30 cycles of 94°C for 30 s, 56°C for 30 s, and 72°C for 30 s with a 5 min extension at 72°C. The primers used were bdlU8-F (5’-AGAGGATGGGCAGTTAGGC-3’) and bdlU8-R (5’-TTTCTGAGGCACAAGGGAGT-3’). A poplar actin (*PeACT*) cDNA amplified using the PeACT-F (5’-GTCCTCTTCCAGCCATCTC-3’) and PeACT-R (5’-TTCGGTCAGCAATACCAGG-3’) primers served as an internal control. PCR products were electrophoresed on a 1.5% agarose gel and viewed under UV light after standard staining with ethidium bromide.

For real-time quantitative RT-PCR analysis, the specific primers DLU8-F (5’-GGATGGAATAAGTTTGGACAGG-3’) and DLU8-R (5’-CCGTTCAGTAACAGCAAGTGTG-3’) were used. The *PeACT* gene was used as a loading control. Fluorescence-quantitative PCR reactions were repeated at least three biological replicates (each repeat, three technical replicates).

### Generation of *PeUVR8*-transgenic Arabidopsis plants

To generate Arabidopsis lines overexpressing *PeUVR8*, the full-length *PeUVR8* sequence was amplified by PCR using the PeUVR8-F and PeUVR8-R primers. The amplified cDNA was cloned into the expression vector pRI (pRI 101-AN) under the control of the cauliflower mosaic virus (CaMV)-35S promoter. The plasmid was then transformed into WT and *uvr8* mutant Arabidopsis lines using the *Agrobacterium* strain GV3101 and the floral dip method. T3 plants from three independent lines were used for analysis.

### Hypocotyl measurements

For hypocotyl growth experiments, the hypocotyl lengths of at least 30 4-day-old Arabidopsis seedlings of each line grown under appropriate conditions (with or without UV-B) were measured: all of these seedlings were put in agar plates to make them straight and measured with caliper. At least three independent biological replicates were performed for all experiments.

### Measurement of total anthocyanin concentration

Total anthocyanin was extracted using the methanol-HCl method. Samples (0.1g) grown under appropriate conditions were extracted overnight in 5-ml methanol and 1% (v/v) HCl at room temperature. The absorbance of each extract was measured at 530, 620, and 650 nm using a UV-1600 spectrophotometer (Shimadzu, Kyoto, Japan). The relative anthocyanin content was determined using the formula OD = (A_530_ –A_620_)– 0.1(A_650_ –A_620_) [51]. One unit of anthocyanin content was defined as a change of 0.1 OD (unit × 10^3^/g fresh weight).

### Bimolecular fluorescence complementation (BiFC) assay

Full-length *PeUVR8* and *AtCOP1* cDNAs were cloned into pSPYNE-35S and pSPYCE-35S vectors, which contain DNA encoding the N- or C-terminal regions of YFP (Y^N^ or Y^C^), respectively, according to previous protocols (Walter et al., 2004). Both the Y^N^ and Y^C^ were located in the C-terminal of the inserted sequences. Onion epidermal cells were transformed transiently using the *Agrobacterium* infection method as described before [52], with different combinations of these constructs. YFP-dependent fluorescence was detected 24 h after transfection under specific UV-B irradiation conditions (1.5 μmol m^–2^ s^–1^ narrowband UV-B) using an LSM 510 Meta confocal laser-scanning microscope (Carl Zeiss).
